# A Machine Learning Method for the Fast Simulation of the Scattering Characteristics of a Target Under a Planar Layered Medium

**DOI:** 10.3390/s25082481

**Published:** 2025-04-15

**Authors:** Zhaoyu Wang, Qinghe Zhang, Zhaoyang Shen, Lei Zhang, Han Liu

**Affiliations:** 1 College of Computer and Information Technology, China Three Gorges University, Yichang 443002, China; 202208100021013@ctgu.edu.cn (Z.W.); shenzhaoyang@ctgu.edu.cn (Z.S.); zhanglei@ctgu.edu.cn (L.Z.); liuh@ctgu.edu.cn (H.L.); 2 Hubei Key Laboratory of Intelligent Vision Based Monitoring for Hydroelectric Engineering, China Three Gorges University, Yichang 443002, China

**Keywords:** ground penetrating radar, planar layered medium, buried target, fast forward simulation, principal component analysis, machine learning

## Abstract

Numerical simulation of ground-penetrating radar (GPR) has been widely used to enhance the interpretation of GPR data and serves as a key component in Full Waveform Inversion (FWI). In response to the time-consuming numerical computation of layered medium and buried targets, which leads to inefficiency in full-wave inversion, this paper proposes a machine learning-based forward scattering rapid solution method. Using the detection of rebar buried in concrete under sand as the GPR application scenario, with scene parameters such as concrete moisture content, rebar radius, and burial depth, scattering echo signals are obtained via Finite Difference Time Domain (FDTD) simulation. Principal component analysis (PCA) is applied to reduce the dimensionality of the echo data, and the first 40 principal component weight coefficients are selected as the output of the deep learning network. An innovative cyclic nested deep learning network architecture is designed, which not only fully explores the intrinsic causal relationship between the scene parameters and the principal component weight coefficients, but also refines and corrects each predicted principal component. The numerical results demonstrate that, compared with traditional machine learning methods, the cyclic nested machine learning network architecture offers higher prediction accuracy and learning efficiency, validating the effectiveness of the proposed method.

## 1. Introduction

In practical physical environments, layered media are widely present in various scenarios, compared to a single uniform medium [1,2]. For example, in areas prone to debris flows, the soil environment is divided into loose soil zones and slightly denser base areas; in lunar exploration, the lunar subsurface structure is divided into regolith and bedrock. In many fields such as underground resource exploration, urban infrastructure construction, and military reconnaissance, accurately acquiring information about buried targets in a layered medium is significant. In oil exploration, understanding the distribution of underground oil layers plays a decisive role in resource extraction decisions; in urban construction, precisely locating buried pipelines, cables, and other facilities can effectively prevent accidental damage during the construction process.

However, traditional forward methods face limitations such as low computational efficiency and limited accuracy when dealing with buried target problems in layered media [3]. Ground-penetrating radar (GPR) is a non-destructive underground detection technique that uses electromagnetic waves [4]. Fast forward simulation techniques are crucial for understanding the underground scattering mechanisms of GPR and for data interpretation inversion. Various forward full-wave solvers have been developed to describe the radar characteristics of underground objects, including the Finite Difference Time Domain (FDTD) method [5], Method of Moments (MoM) [6], and Finite Element Time Domain (FETD) method [7]. However, these physics-based traditional solvers typically require substantial computational time, especially when signal processing or machine learning-based inversion algorithms need to be repeatedly executed. When Full Waveform Inversion (FWI) adopts global optimization [8], objective function gradient solving requires hundreds of forward problem solving techniques. As a result, the inversion of each GPR dataset often requires hundreds or even thousands of iterations [9].

In recent years, Machine Learning (ML) [10] has become an attractive choice for solving GPR and other electromagnetic problems, aiming to alleviate the computational burden of traditional GPR forward solvers. These machine learning methods utilize Artificial Neural Networks (ANNs) to construct models and train algorithms using provided training data. For example, neural networks have been used to detect hyperbolic features in B-scan scans [11], fully connected feedforward neural networks have been employed for landmine detection and classification, and Convolutional Neural Networks (CNNs) have also been used for object detection in GPR profiles [12,13]. Some researchers have proposed a coupled NN-RF method for estimating the diameter of rebar in concrete [14] and introduced a fast ML forward solver. The authors of [15] describe the development of a machine learning-based fast forward solver for concrete slab GPR data, which utilizes dense networks and principal component analysis (PCA) to predict the A-scan response of the target below the antenna center. The forward solver is used to estimate the moisture content of concrete and the depth and diameter of rebar. As part of Full-Wave Inversion (FWI), this approach validated the accuracy of using ML models as forward solvers. Wang Haoyu proposed a Full-Waveform Inversion method based on Overall Coding Genetic Algorithms (OCGAs) [16], which improves the encoding strategy based on genetic algorithms, encoding the overall features of the individual. The above research mainly focuses on half-space and buried target detection and does not address the scenario of layered media and buried targets, with the input dimension of the designed machine learning network being relatively large, which reduces the prediction accuracy and limits the effective application of the method.

This paper addresses the rapid simulation of scattering characteristics for buried targets in planar layered media, focusing on the practical application of ground-penetrating radar (GPR) to detect rebar embedded in concrete beneath sand. The model parameters include the moisture content of the concrete, the radius of the rebar, and the burial depth. Scattering echo signals are obtained through simulation. Subsequently, a principal component analysis (PCA) is employed for dimensionality reduction in the echo data. The analysis reveals that only the first 40 principal components’ weight coefficients are necessary to achieve a good fit for the original simulated echo data, thereby creating a suitable dataset format for subsequent network training. Additionally, an innovative nested loop structure machine learning approach is proposed. Various machine learning methods are applied, including single hidden layer neural networks, particle swarm-optimized single hidden layer neural networks, double hidden layer neural networks, and random forest methods. These techniques are combined with the nested loop structure and compared in terms of accuracy and speed. This comparative study aims to thoroughly validate the precision and efficiency of the proposed method in the forward modeling of buried targets in planar layered media.

## 2. Materials and Methods

**Figure 1:**
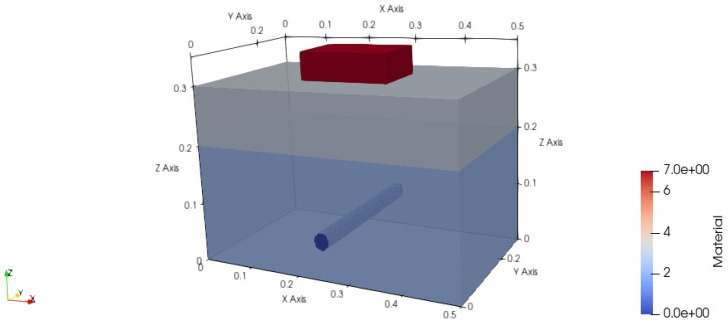
GPR planar layered medium model structure. (The antenna model is within the red box, and the cylinder represents the PEC rebar.).

The application scenario of the planar layered medium model in this paper was set to a rebar buried in concrete under sand, as shown in Figure 1. The size of the detection area was 0.5×0.3×0.4 meters. The first layer was sand with a depth of 0.1 m, and the second layer was concrete with a depth of 0.2 m, with the detection target, the rebar, buried in the second layer. The rebar was modeled as a perfect electric conductor (PEC). There was no air region between the perfect matched layer (PML) and the target. This is because, when simulating the propagation of electromagnetic waves in a certain medium, and only focusing on the field distribution inside the medium, the PML is directly set at the boundary of the medium.

In all simulations, the antenna’s main axis was parallel to the rebar’s main axis.

The dielectric properties of concrete, as a dispersive medium, are described by the extended Debye model [17]:

(1) $$\varepsilon =\varepsilon_{\infty }+\frac{\varepsilon_{s}-\varepsilon_{\infty }}{1+j\omega t_{0}}+\frac{\sigma }{j\omega \varepsilon_{0}},$$

In the equation, $\varepsilon_{\infty }$ is the relative permittivity at infinite frequency, $\varepsilon_{S}$ is the relative permittivity at zero frequency, $t_{0}$ is the relaxation time, σ is the conductivity, ω is the angular frequency, $j=\sqrt{-1}$, and $\varepsilon_{0}$ is the permittivity of free space. Due to the complex geometric structure of the detection scene, the same model with unified parameters was constructed in the GPRMAX (v3.1.7) software based on the FDTD principle, and the simulated time-domain waveform was compared and analyzed. This method can not only reduce the error caused by model differences, but also improve the reliability of the results, and further verify the results of FDTD.

The GPR forward problem was numerically simulated using the FDTD method to obtain A-scans. Considering computational resource limitations, the spatial step size was determined based on stability and numerical dispersion conditions, with the spatial discretization set to 1 cubic millimeter and the time step set to the Courant limit. The GPR antenna in the FDTD simulation used a 1.5 GHz antenna model from Geophysical Survey Systems, Inc. (GSSI) (with a minimum resolution of 4 mm), polarized parallel to the rebar’s main axis. This model was originally created by Warren and Giannopoulos [18], where the antenna’s geometry was measured, and then the unknown dielectric properties of the antenna were derived using Taguchi optimization. This updated and improved 1.5 GHz GSSI model was used in the FDTD simulation to generate training data. The incident wave commonly used was a Gaussian pulse wave. The Gaussian pulse wave has the characteristic of a wide frequency band and can effectively simulate the short pulse signals emitted by an actual ground-penetrating radar system. This makes it highly suitable for detecting the structure and properties of subsurface media. The rebar in concrete was modeled as a Perfect Electric Conductor (PEC) cylinder, with a radius varying from 5 to 29.5 mm and a burial depth ranging from 0 to 200 mm. In this study, the relative permittivity, conductivity, permeability, and magnetic loss values for the sand were set to 6, 0, 1, and 0, respectively. These parameters provide a foundational quantitative basis for subsequent research on the characteristics of sand in electromagnetic environments and the construction and analysis of related models.

## 3. Network Architecture and Learning Strategy

Machine learning, as an important branch of artificial intelligence, plays a decisive role in model performance based on the choice of network architecture and learning strategy. The neural network architecture and learning strategy designed and utilized in this paper combined traditional machine learning techniques, and through a comparative analysis of accuracy and speed, we deeply explored their application in the fast forward simulation of underground targets in a planar layered medium. In the forward problem, the inputs included concrete moisture content (WC), rebar radius (R), and rebar burial depth (D).

The design philosophy of the proposed network architecture and learning strategy was to utilize the interdependence between the 40 principal component weight coefficients obtained through PCA dimensionality reduction, where the previous principal component weight coefficient should help predict the next one. Through this strategy, the predicted principal component weight coefficients can be obtained. By multiplying the predicted principal component weight coefficients with the corresponding principal component eigenvectors, the effective fitting of the A-scans echo data can be achieved.

**Figure 2:**
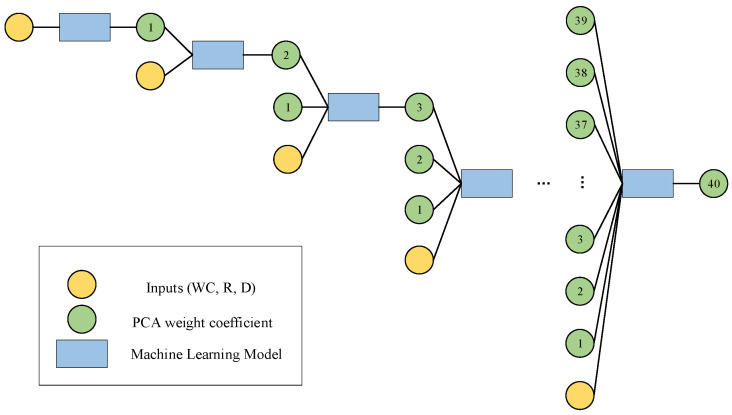
Architecture and learning strategy of the first part of the network.

The nested loop neural architecture proposed in this paper consisted of two independent network architectures. For the first network architecture, the first step was to input a vector composed of model parameters, including moisture content (WC), radius (R), and depth (D), in order to predict the first principal component. Then, the model parameters were combined with the first predicted principal component to predict the second principal component. The causal relationship between these principal components not only constrains the range of the prediction results but also effectively reduces the optimization search space, enhancing both computational efficiency and model performance. The second step involved using the model parameters along with the previously predicted principal components (the first and second principal components) to predict the third principal component. This process continued until all forty principal components were predicted. It is important to note that each stage was independently trained, effectively avoiding the common issues of gradient vanishing and overfitting in deep architectures, ensuring the stability and reliability of the model. At each stage, the data were re-divided into training, validation, and test sets, a strategy that ensured the generalization ability of the neural network, prevented over-reliance on a specific dataset, and improved the model’s adaptability and robustness. Figure 2 shows the network structure and learning strategy of the first part, forming a deep learning system consisting of 40 layers.

**Figure 3:**
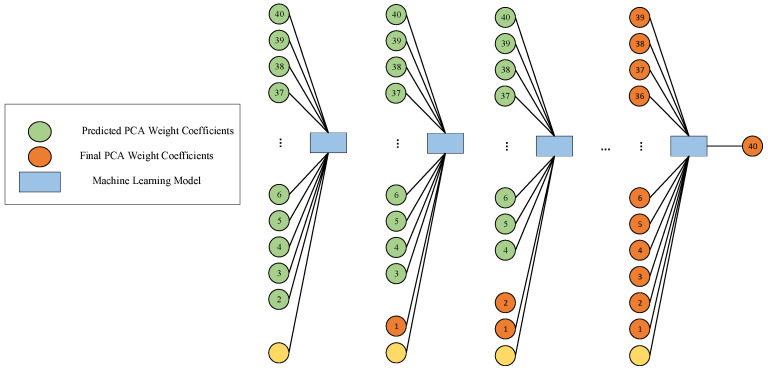
Architecture and learning strategy of the second part of the network.

However, due to the inherent uncertainty in the prediction process, these coefficients inevitably exhibit a certain degree of bias. To address this issue, a subsequent module was designed and introduced, with the core goal of establishing the causal relationship between the error of the predicted values relative to the actual axis and the model parameters, with a particular focus on the analysis of the differences between the predicted values and the actual axis. For the second network architecture, the first step was to combine the model parameters with all the principal components except the first predicted principal component to predict the corrected first principal component. The second step involved using the model parameters along with the corrected first principal component and all the principal components except the second to predict the corrected second principal component. This structure was iteratively executed until all components were fully corrected, forming a complete and corrected set of principal components. Figure 3 visually illustrates the second part of the architecture, closely integrating with the first part of the network architecture.

Similar to the first part of the architecture, the second part adopted independent training, using model parameters, the output of the initial module, and the output from the previous stage of the subsequent module as inputs, ensuring the independence and accuracy of the training process, with the final PCA weight coefficients as the output. At each stage, the original data were scientifically divided into training, validation, and test sets. This strategy further reduced the risk of overfitting and significantly enhanced the model’s generalization ability and robustness. The final output was a nearly real-time (≈1 s) solver for the 40 principal components, which can be decompressed to provide GPR A-scans under predicted time-domain modeling. It is worth noting that the designed neural network innovatively uses the predicted principal components as inputs, rather than the actual principal components. This design simplifies the network input, requiring only the provision of key parameters such as concrete moisture content, rebar radius, and burial depth.

## 4. Data Acquisition and Processing

To obtain the dataset required for the machine learning network, 2000 different scenarios were set up, and spline interpolation was used to obtain the Debye properties corresponding to moisture contents ranging from 0.2% to 12%. These properties were used as inputs in the FDTD algorithm to simulate concrete, generating a dataset of 2000 3D GPR models for rebar buried in concrete, which was then used to train the ML model. For each model, moisture content (WC), radius (R), and depth (D) were randomly and uniformly distributed within their respective ranges:

(1) WC∈[0,12]%.
(2) R∈[5,29.5]mm.
(3) D∈[0,200]mm.

Any regression scheme using three input parameters to predict a relatively large vector increases the complexity of the problem and introduces instability during training, leading to noisy outputs. This can be partially mitigated by down sampling the traces every 10 time steps. To further reduce the dimensionality of the data, a principal component analysis (PCA) was employed.

PCA [19], as one of the methods for dimensionality reduction, achieves this by increasing interpretability while minimizing information loss. It does so by creating new uncorrelated variables that maximize variance consecutively. These new variables are called principal components. The process of finding these principal components simplifies to solving an eigenvalue problem, and the new variables are defined by the dataset at hand, rather than being a priori, making PCA an adaptive data analysis technique. Reference [20] proposed an automatic mode selection scheme based on mode decomposition and principal component analysis for GPR signal denoising.

**Figure 4:**
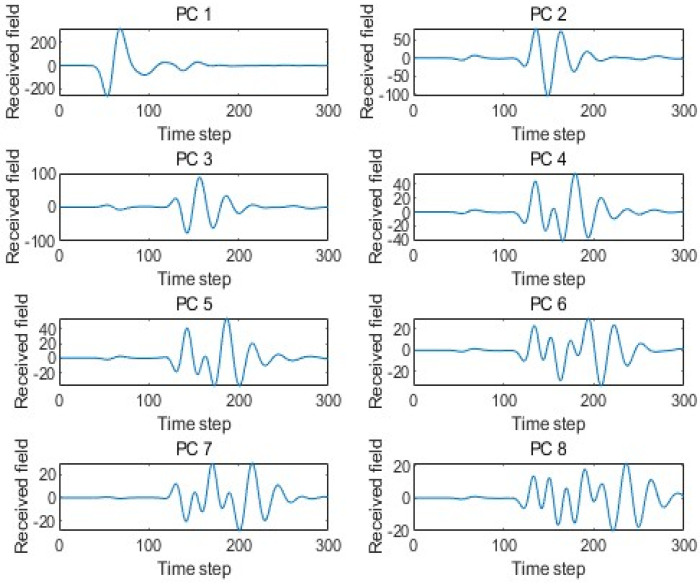
The eight dominant principal components of the training set.

PCA maps the data onto its principal orthogonal axes, and the data can be reconstructed through their linear combinations. The principal axes of the training set are composed of 300 eigenvectors. The linear combination of these 300 eigenvectors can reproduce each dataset in the training set. Figure 4 shows eight arbitrary principal components out of the 300 eigenvectors in the sample data under the scenario model.

To reduce the dimensionality of the training set, this paper did not use all 300 principal components obtained from PCA as sample data. In fact, a linear combination of a certain number of principal components can accurately fit the echo data in the training set. Echo data refer to the data reflected back after the signal encounters obstacles or changes in the medium during transmission. Many systems (such as radar and sonar) rely on the echo signal, and the time domain can clearly capture the details of the echo: determine the location of the target and reflect the material or size of the target.

**Figure 5:**
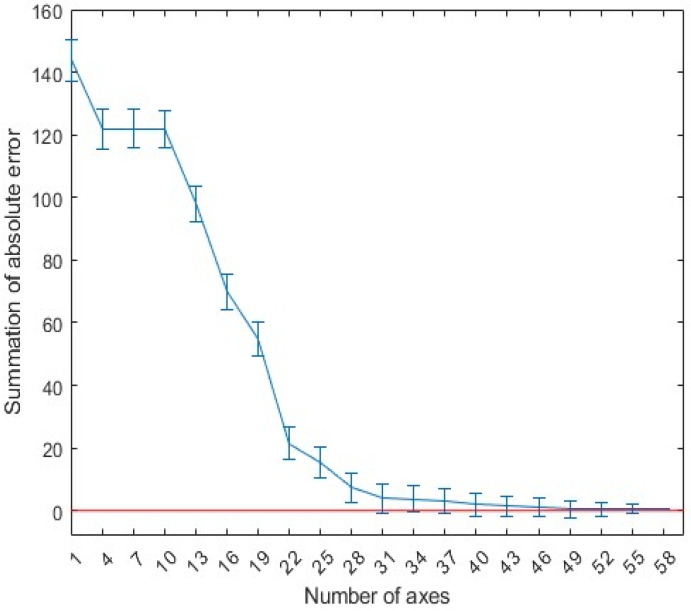
Average error between the actual and compressed A-scans (error bars correspond to one standard deviation).

Figure 5 shows the absolute error of the linear fit of the echo data using the first 40 principal components. In this figure, it can be seen that, when the first 40 most significant principal components are used for fitting, the original echo can be approximated very accurately. Therefore, the first 40 out of the 300 eigenvectors were selected as the principal components of the GPR scattering echo. This approach ensures that no information contained in the neglected principal components is lost, while also further reduces the dimensionality to speed up the network’s training time.

The compressed version of these eigenvectors is then calculated as:

(2) Y=Aω,

where Y is the vector representing the actual trajectory, A is the matrix containing the 40 principal axes of the training set, and ω is the vector containing the 40 principal component weight coefficients. The vector ω can be computed using the least squares method, $\omega ={\left(A^{⊤}A\right)}^{-1}A^{⊤}Y$. Therefore, all trajectories can be reproduced using the matrix A and their unique principal component weight coefficients ω. These weight coefficients ω were used as the output of the machine learning network in the subsequent GPR forward simulation, while the model parameters (WC,R,D) served as the input.

**Figure 6:**
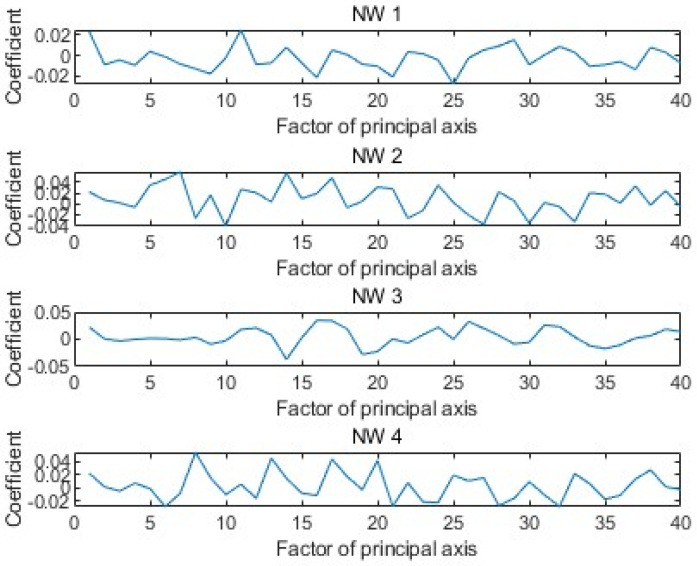
The weight coefficients of the 40 principal components used to represent the compressed signal.

**Figure 7:**
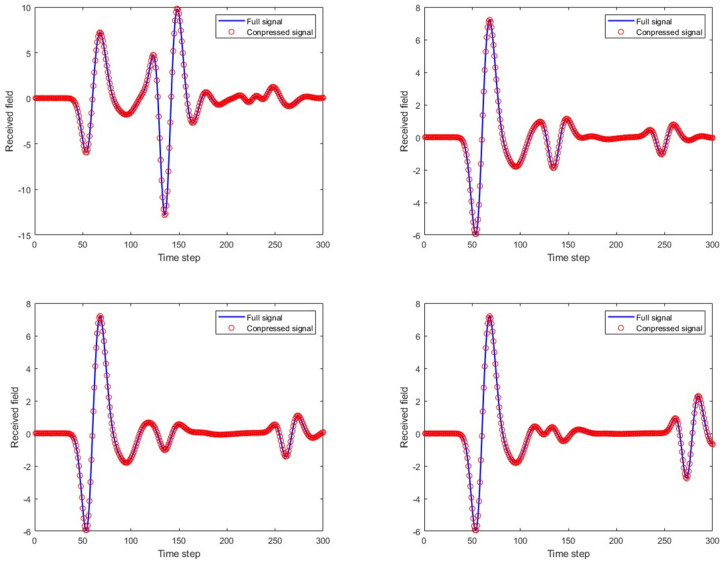
Four complete A-scan scans calculated by FDTD and their corresponding compressed PCA representation using 40 principal axes.

Figure 6 shows the weight coefficients corresponding to the first 40 principal components of the GPR echo data under four arbitrary model parameters. Figure 7 compares the full A-scans echo data from four FDTD numerical simulations with the echo data obtained through linear fitting of the 40 principal components. In the figure, it can be seen that the match between the two is good, providing a solid theoretical basis for the subsequent rapid GPR forward simulation.

## 5. Results and Analysis

The dataset in this paper included 2000 randomly generated scenarios and their corresponding simulated echo data. The dataset was divided into training set (70%), validation set (15%), and test set (15%). The input to the network model consisted of an input vector formed by the concrete moisture content (WC), rebar radius (R), and rebar burial depth (D), while the output vector corresponded to the principal component weight coefficients. The final output vector was multiplied by the principal component eigenvectors to achieve the fitting of the A-scan echo data.

**Figure 8:**
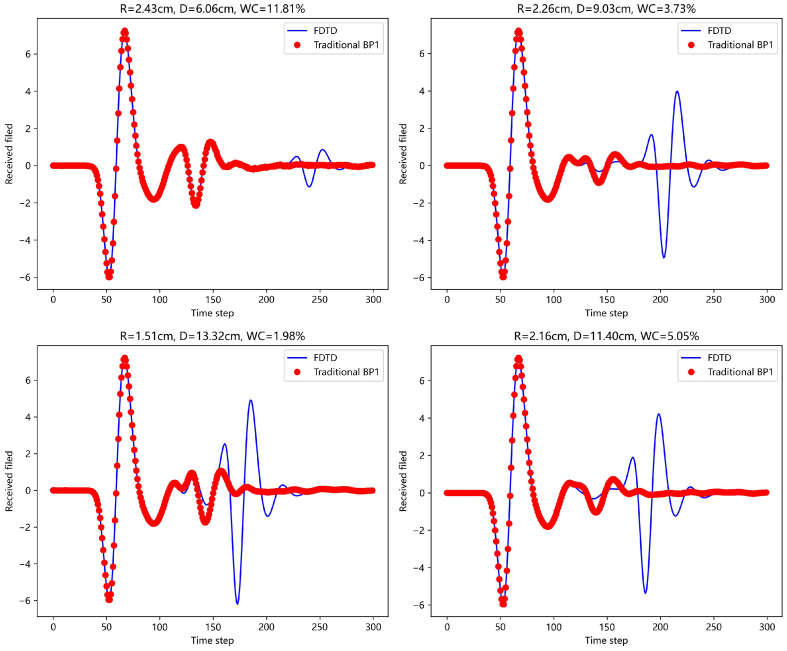
Under the four model parameters, the prediction results of traditional BP network (single hidden layer) are compared with those of FDTD.

**Figure 9:**
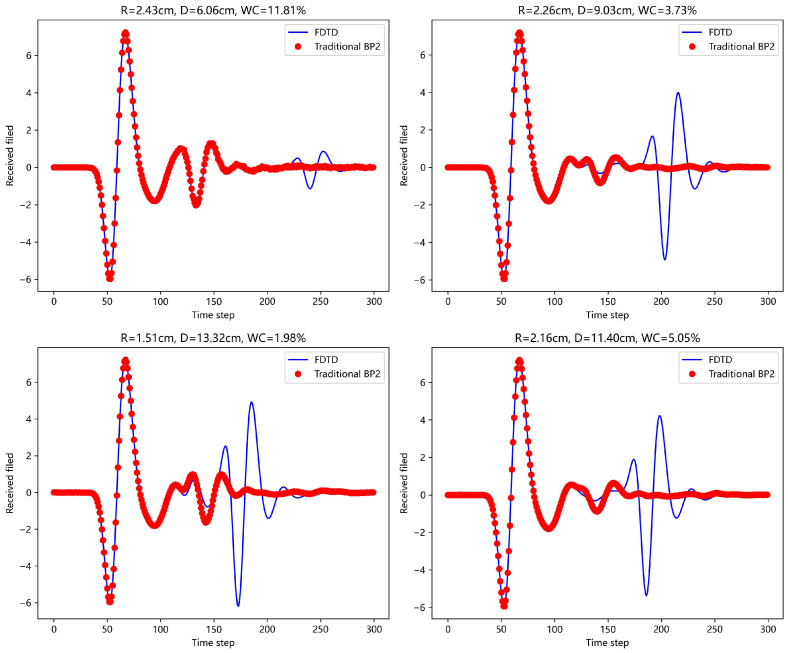
Under the four model parameters, the prediction results of traditional BP network (dual hidden layers) are compared with those of FDTD.

First, a traditional BP neural network (with single and double hidden layers) was used to predict the echo signals. Figure 8 and Figure 9 show the prediction results for four different scenarios and compare them with the actual FDTD simulation results. In the figures, it is clear that the prediction is good for the first A-scan layer, but fails to provide accurate predictions for the concrete layer. The complex physical properties of the concrete layer make it difficult for the traditional BP network to avoid error accumulation during the learning process, leading to poor forward simulation results for the concrete layer.

**Figure 10:**
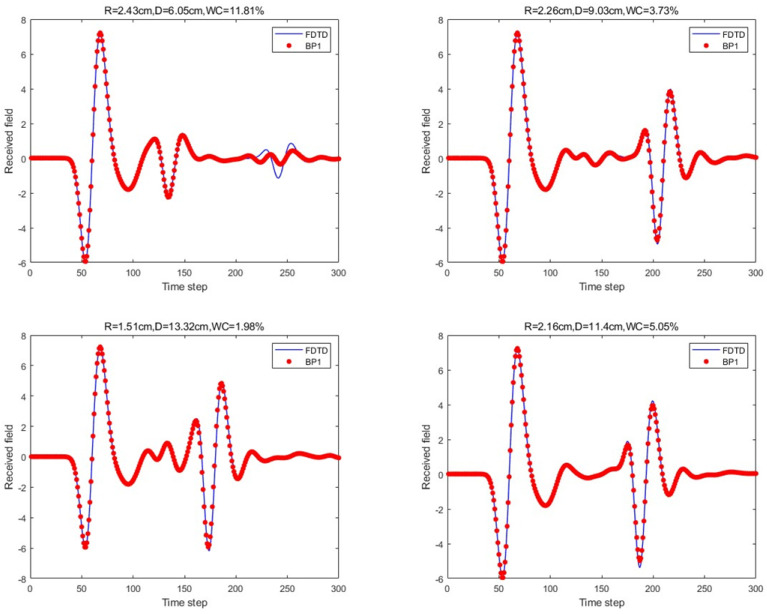
Under the four model parameters, the prediction results of the proposed method (single hidden layer) are compared with those of FDTD.

Next, the scattering echoes were predicted using the method proposed in this paper. The machine learning model in the nested loop network architecture was the traditional BP neural network. The network structure included an input layer, one hidden layer, and an output layer. The hidden layer contained 20 neurons, and the ReLU activation function was used to introduce nonlinearity. The mean square error (RMSE) was used as the loss function. For the same four scenario parameters mentioned earlier, the prediction results are shown in Figure 10.

**Figure 11:**
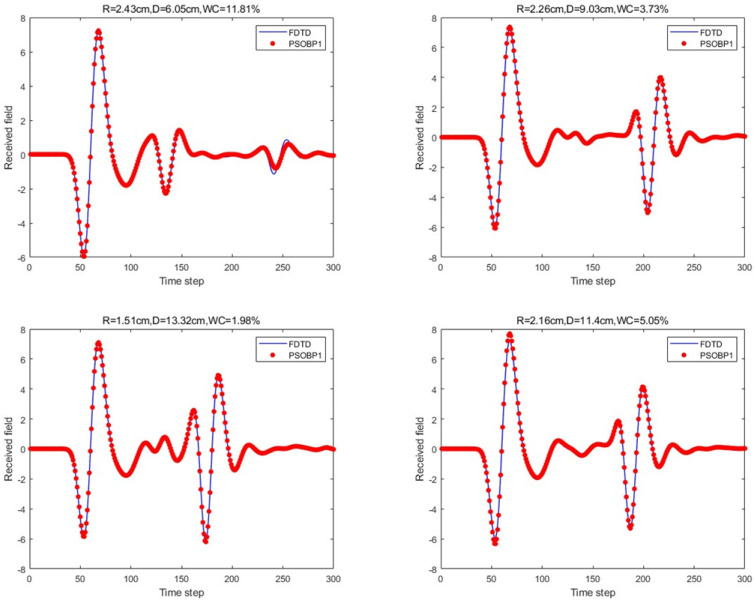
Under the four model parameters, the prediction results of the proposed method (PSO-optimized single hidden layer) are compared with those of FDTD.

Next, for the BP neural network with a single hidden layer in the aforementioned network architecture, the Particle Swarm Optimization (PSO) [21] Algorithm was introduced. The PSO algorithm treats the weights and biases of the BP neural network as the positions of particles and simulates the flight behavior of the particle swarm to search for the optimal combination of weights w and biases b in the search space. The learning factors were set as c1 = c2 = 2, the number of population updates was 30, the population size was 5, and the velocity range was [−1, 1], with a boundary range of [−1, 1]. The prediction results for the four scenario scattering echoes are shown in Figure 11.

**Figure 12:**
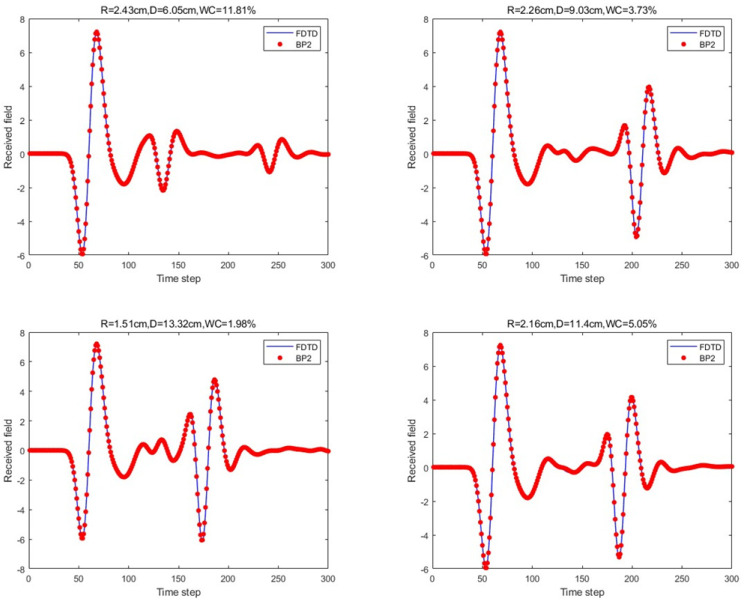
Under the four model parameters, the prediction results of the proposed method (dual hidden layers) are compared with those of FDTD.

Finally, the BP neural network with two hidden layers in the proposed network architecture was considered. The two hidden layers consisted of 20 and 15 neurons. The prediction results for the four scenario scattering echoes are shown in Figure 12.

For the scattering echo prediction study under four scenarios using five different methods, as shown in Figure 8, Figure 9, Figure 10, Figure 11 and Figure 12, the traditional BP neural network fails to accurately predict the subsequent echo data, resulting in significant errors. However, when the BP neural network is combined with the network architecture proposed in this paper, the results show significant improvement. Despite this, the single hidden layer may exhibit considerable deviation for different cases; thus, PSO optimization was used, leading to a noticeable enhancement in prediction accuracy. Subsequently, the A-scans obtained from the BP neural network with dual hidden layers for rapid GPR forward simulation were compared with the A-scans directly obtained from the FDTD forward solver, and the results were nearly identical. Table 1 shows the mean square error analysis of the scattering echo prediction results for the four scenarios using the five methods.

In terms of training time, the traditional BP neural network method takes up to 2 h for training. In contrast, the PSO-optimized single hidden layer method proposed in this paper reduces the training time to about 35 min; the dual hidden layer BP network requires 100 s for training, while the single hidden layer BP network trains in the fastest time of 20 s. A GPR forward simulation using the FDTD-based numerical simulation software GPRMAX for a single set of model parameters takes approximately 45 min. In comparison, once trained, the machine learning network constructed in this study can predict A-scans in real time within 1 s, significantly improving prediction efficiency.

## 6. Conclusions

This paper proposes a new method for GPR simulation in a layered medium, which serves as an alternative to traditional methods such as FDTD. The method is based on a neural network regression approach that employs an innovative nested loop structure combined with a BP neural network with dual hidden layers. The input to the network model consists of scene parameters (concrete moisture content, rebar radius, and burial depth), and the output is the PCA weight coefficients of the scattering echo. The output is then multiplied by the principal component eigenvectors to achieve the fitting and prediction of the A-scan echo data. The numerical results show that the proposed nested loop machine learning network architecture offers a higher prediction accuracy and learning efficiency.

## Figures and Tables

**Table 1:** Mean square error of five methods across four cases.

Method	Model 1	Model 2	Model 3	Model 4
**Traditional BP1**	0.23363	1.00906	1.22479	1.09911
**Traditional BP2**	0.23095	1.00898	1.22437	1.08582
**BP1**	0.03216	0.01215	0.03516	0.01947
**PSO_BP1**	0.01282	0.00336	0.00170	0.01347
**BP2**	0.00052	0.00099	0.00605	0.00171

Notes: Traditional BP1: the single hidden layer BP neural network. Traditional BP2: the dual hidden layer BP neural network. BP1: the nested loop machine learning network collaborates with the single hidden layer BP neural network. PSO_BP1: the nested loop machine learning network collaborates with the PSO-optimized single hidden layer BP neural network. BP2: the nested loop machine learning network collaborates with the dual hidden layer BP neural network.

## Data Availability

The data underlying the results presented in this paper are not publicly available at this time but may be obtained from the authors upon reasonable request.

## References

[1] Zhang J. (2021). Research on the Propagation Characteristics of Vortex Electromagnetic Waves in Layered Media. Master’s Thesis.

[2] Yamauchi Y., Kidera S. Inverse scattering enhanced synthetic aperture imaging for multi-layered ground media. Proceedings of the International Symposium on Antennas and Propagation (ISAP).

[3] Savita S.J., Pallavi A. Modeling of GPR using gprMax Simulation. Proceedings of the 2022 IEEE International Conference on Distributed Computing and Electrical Circuits and Electronics (ICDCECE).

[4] Alioua C., Grimes M., Kemache N., Benredjiem O., Roula R. Locating and estimating the depth of a buried cable using GPR-Radar and convolutional neural networks. Proceedings of the 2024 2nd International Conference on Electrical Engineering and Automatic Control (ICEEAC).

[5] Zhao S., Wei B., He X., Deng H. (2023). 2-D hybrid cylindrical fdtd method with unconditional stability. IEEE Microw. Wirel. Technol. Lett..

[6] Araújo A.R.J.d., Colqui J.S.L., Azevedo W.L.M.d., Kurokawa S., Filho J.P., Kordi B. (2022). Transient analysis of grounding electrodes in multilayer soils using method of moments. IEEE Lat. Am. Trans..

[7] Kim J., Teixeira F.L. On the parallel performance of SPAI-FETD solvers for time-domain maxwell’s equations. Proceedings of the 2022 United States National Committee of URSI National Radio Science Meeting (USNC-URSI NRSM).

[8] Ren Y., Xu X., Yang S., Nie L., Chen Y. (2020). A physics-based neural-network way to perform seismic full waveform inversion. IEEE Access.

[9] Feng D., Cao C., Wang X. (2019). Multiscale full-waveform dual-parameter inversion based on total variation regularization to on-ground GPR data. IEEE Trans. Geosci. Remote Sens..

[10] Akhaury U., Giannakis I., Warren C., Giannopoulos A. Machine learning based forward solver: An automatic framework in gprMax. Proceedings of the 2021 11th International Workshop on Advanced Ground Penetrating Radar (IWAGPR).

[11] Wang B., Chen P., Zhang G. (2023). Simulation of GPR b-scan data based on dense generative adversarial network. IEEE J. Sel. Top. Appl. Earth Obs. Remote Sens..

[12] Liu Q., Zhu C. Automatic detection for road voids from GPR images using deep learning method. Proceedings of the 2023 4th International Conference on Computer Vision, Image and Deep Learning (CVIDL).

[13] Akçali S., Erden F. Support of data augmentation with GAN on faster R-CNN based buried target detection. Proceedings of the 2021 29th Signal Processing and Communications Applications Conference (SIU).

[14] Giannakis I., Giannopoulos A., Warren C. (2021). A machine learning scheme for estimating the diameter of reinforcing bars using ground penetrating radar. IEEE Geosci. Remote Sens. Lett..

[15] Giannakis I., Giannopoulos A., Warren C. (2019). A machine learning-based fast-forward solver for ground penetrating radar with application to full-waveform inversion. IEEE Trans. Geosci. Remote Sens..

[16] Wang H., Liu H., Ma Z., Du P. (2022). Full waveform inversion of ground-penetrating radar based on the overall encoding genetic algorithm. J. Microw..

[17] Balictsis C.M. Saddle point-based description of the propagating pulse dynamics in skin tissues and phantoms represented by Cole-Cole and Debye models. Proceedings of the 2023 IEEE MTT-S International Microwave Biomedical Conference (IMBioC).

[18] Warren C., Giannopoulos A. Investigation of the directivity of a commercial ground-penetrating radar antenna using a finite-difference time-domain antenna model. Proceedings of the 2012 14th International Conference on Ground Penetrating Radar (GPR).

[19] Bhyregowda P., Masum M., Mamudu L., Chowdhurv M., Kosaraiu S.C., Shahriar H. Active PCA: A novel framework integrating PCA and active machine learning for efficient dimension reduction. Proceedings of the 2024 IEEE 48th Annual Computers, Software, and Applications Conference (COMPSAC).

[20] Hao T., Jing L., He W. (2023). An automated GPR signal denoising scheme based on mode decomposition and principal component analysis. IEEE Geosci. Remote Sens. Lett..

[21] Amzar H., Haziq M.I., May Z. Hybrid of PSO-ANN and PCA-SVR models for the prediction of external corrosion in pipeline infrastructure: A comparative study. Proceedings of the 2023 IEEE International Conference on Sensors and Nanotechnology (SEN-NANO).

